# Liver Metastases and Survival Among Patients With Colorectal Cancer

**DOI:** 10.1001/jamanetworkopen.2025.50467

**Published:** 2025-12-22

**Authors:** Ida Ravnsbæk Johannsen, Anders Kindberg Boysen, Mette Fugleberg Nielsen, Frank Viborg Mortensen, Jakob Kirkegård

**Affiliations:** 1 Department of Surgery, Hepato-Pancreato-Biliary Section, Aarhus University Hospital, Aarhus, Denmark; 2 Department of Clinical Medicine, Aarhus University, Aarhus, Denmark; 3 Department of Oncology, Aarhus University Hospital, Aarhus, Denmark

## Abstract

**Importance:**

Colorectal liver metastases (CRLM) are a major cause of death in patients with colorectal cancer (CRC). Understanding patterns of incidence and survival is important to inform clinical care and policy.

**Objective:**

To examine temporal patterns of incidence, characteristics, and survival among patients with synchronous and metachronous CRLM.

**Design, Setting, and Participants:**

This population-based cohort study in Denmark used Danish national health registries, including the Cancer Registry, National Patient Registry, and National Pathology Registry, as data sources. A nested medical record review of a subset of patients (n = 954) was also conducted to assess concordance in incidence between registry data and medical records. All patients diagnosed with CRLM in Denmark between 2007 and 2024 were included.

**Main Outcomes and Measures:**

The end points were the cumulative incidence of CRLM from the date of CRC diagnosis (calculated at 1, 3, and 5 years of follow-up) and overall survival from the date of CRLM diagnosis (estimated using Kaplan-Meier curves and Cox proportional hazards regression models). Hazard ratios with 95% CIs were used to compare survival across diagnosis periods and CRLM subtypes (synchronous, early metachronous, and late metachronous).

**Results:**

A total of 14 785 patients with CRLM (median [IQR] age, 68.9 [62.2-77.0] years; 8616 males [58.3%]) were identified. The 5-year cumulative incidence of CRLM in patients with CRC was 19.8% (95% CI, 19.5%-20.1%). The cumulative incidence of CRLM decreased with age but was highest in patients younger than 50 years (5-year follow-up: 27.2%; 95% CI, 25.6%-28.8%); it was also highest in male patients (5-year follow-up: 21.5%; 95% CI, 21.1%-22.0%). The medical record review showed 26.2% (250 of 954) of patients developed CRLM during the follow-up period. One-year survival increased for patients with synchronous CRLM from 47.2% (95% CI, 45.3%-49.1%) in the 2007-2010 diagnosis period to 58.3% (95% CI, 54.1%-62.3%) in the 2020-2024 diagnosis period. Five-year survival also improved for patients with synchronous CRLM from 13.0% (95% CI, 11.7%-14.3%) in the 2007-2010 period to 20.7% (95% CI, 18.1%-23.5%) in the 2020-2024 period. No improvement was observed for either early or late metachronous CRLM.

**Conclusions and Relevance:**

In this cohort study, over time the cumulative incidence of CRLM decreased across all age groups, while survival increased for patients with synchronous and early metachronous CRLM but not for patients with late metachronous disease. These findings may reflect differences in tumor biology, comorbidity burden, and treatment eligibility and may support targeted follow-up and treatment strategies based on the timing of metastasis.

## Key Points

**Question:**

How has the incidence, characteristics, and survival of colorectal liver metastases (CRLM) changed over time in Denmark?

**Findings:**

In this cohort study of 14 785 patients with CRLM, the cumulative incidence of CRLM decreased across all age groups over time. Survival increased for patients with synchronous CRLM and early metachronous CRLM but not for those with late metachronous CRLM.

**Meaning:**

These results support the need for targeted follow-up and treatment strategies based on the timing of metastasis.

## Teaser

This cohort study evaluates incidence and survival rates of synchronous and metachronous colorectal liver metastases from 2007 to 2024 in Denmark.

## Introduction

Colorectal cancer (CRC) is the third most common cancer in the Western world, with more than 4500 new cases diagnosed annually in Denmark alone.^1,2^ Approximately 25% to 30% of patients with CRC develop liver metastases (colorectal liver metastases [CRLM]),^3,4^ the leading cause of death in patients with CRC.^5^ Liver metastases may develop either as synchronous or metachronous. Synchronous CRLM are generally defined as liver metastases diagnosed concurrently with, or shortly after, the primary CRC.^6,7^ In contrast, metachronous CRLM lacks a consistent definition, with intervals ranging from 1 to more than 12 months following CRC diagnosis.^8,9,10,11^ The lack of agreement on the definition of metachronous CRLM impairs comparisons between studies. A 2023 European consensus project proposed defining synchronous CRLM as metastases diagnosed concurrently with the primary tumor, while classifying metachronous CRLM into early (within 12 months of CRC diagnosis) and late (more than 12 months after CRC diagnosis).^12^ Although the global burden of CRC is increasing, nationwide screening programs may have contributed to a lower incidence of CRLMs. Robust population-based studies are crucial for generating reliable data on incidence, treatment patterns, and survival in large cohorts, enabling a more representative assessment of therapeutic effectiveness and shifts in patient outcomes. In the present study, we aimed to examine temporal patterns of incidence, characteristics, and survival among patients with synchronous and metachronous CRLM. We also sought to evaluate the accuracy of the incidence of CRLM in the Danish national registries by conducting a medical record review of a random sample of the population.

## Methods

The Danish Data Protection Agency approved this cohort study, and the Central Denmark Region approved the medical record review. No ethical approval or patient consent is required for register-based studies in Denmark. We followed the Strengthening the Reporting of Observational Studies in Epidemiology (STROBE) reporting guideline.

### Setting, Design, and Data Sources

We conducted a nationwide, population-based cohort study of all patients in Denmark diagnosed with CRC from January 1, 2007, to December 31, 2021, using information from the Danish Cancer Registry (DCR), the Danish National Patient Registry (DNPR), and the Danish National Pathology Registry (DPR). The patients were eligible for inclusion if they also had a diagnosis of CRLM from January 1, 2007, to March 8, 2024, recorded in one of the aforementioned databases or in the Danish Liver Cancer Database (DLCD). The registries can be linked at the individual-level using the Civil Personal Registration number, which is assigned to all Danish citizens at time of birth or immigration.^13^ Administrative data on birth date, sex, vital status, and migration have been registered in the Civil Registration System for every Danish resident since 1968.^13^ It is updated daily and virtually complete.

Since being established in 1943, the DCR has contained information on cancer site, date of diagnosis, dissemination, histologic type, and other clinical variables for all cancers diagnosed in Denmark.^14^ The DNPR has information on all inpatient hospitalizations in public hospitals in Denmark since 1977. Since 1995, outpatient and emergency department visits have been included.^15^ Surgical procedures have been registered since 1977 and recorded according to the *Nordic Medico-Statistical Committee Classification of Surgical Procedures*.^16^ Oncological treatment has been registered since 2004.

The Danish Colorectal Cancer Group Database (DCCD) contains information on all patients with colon cancer (since 2001) and rectal cancer (since 1994) diagnosed and/or treated at a surgical department since 1994. The database provides information on treatments; pathology; patient characteristics; and risk factors such as smoking, alcohol consumption, and body mass index.^17^ Data from the DCCD were available through 2020. The DLCD was established in 2014 and contains information on size and number of CRLM. Data on treatment and resection margins are also available among other surgical variables. Until 2024, the DLCD exclusively included information on patients treated with curative intent. Since 2024, patients receiving palliative chemotherapy and best supportive care have been included in the database. The DPR has maintained information on all tissue examinations and pathology specimens from Danish hospitals since 1997.^18^

### Study Population

We identified all patients, aged 18 years or older, with a diagnosis of CRC in the DCR or DNPR from January 1, 2007, to December 31, 2021, as the source population. In case of different dates of diagnoses between the DCR and DNPR, we prioritized the dates from the DCR (eFigure 1 in Supplement 1). From this source population, we identified all patients with information compatible with a diagnosis of CRLM from January 1, 2007, to March 8, 2024, using information from the DNPR, DPR, DCR, and DLCD (eFigure 1 in Supplement 1). Patients were grouped into periods based on year of CRC diagnosis: 2007 to 2010, 2011 to 2014, 2015 to 2018, or 2019 to 2021.

From the source population of 72 722 patients, we randomly sampled 1000 patients stratified by age group, sex, period of diagnosis, and treating hospital in the Central Denmark Region. Due to an insufficient number of patients in some of the strata, the final sample was 954 patients. We conducted a medical record review of these patients, collecting information on date and location of CRC metastases. The review was initiated in February 12, 2024, and completed on March 8, 2024.

### Tumor Characteristics and CRLM Definition 

Information on tumor stage at time of CRC diagnosis was retrieved from the DCR, DNPR, and DCCD. TNM classification was used to record tumor stage (TNM stage) according to the Union of International Cancer Control [UICC]’s *TNM Classification of Malignant Tumours* edition at the time of diagnosis (6th edition until 2017; 8th edition thereafter^19,20^). Tumor stage (UICC stage^19^) was derived from the TNM stage. From the DCR and DPR, we obtained information on tumor histologic type (adenocarcinoma, mucinous adenocarcinoma, signet ring cell carcinoma, and other [including, but not limited to, pleomorphic carcinoma, sarcomatoid carcinoma, and stromal tumor]) and CRC location (right-sided colon, transverse colon, left-sided colon, or rectum).

CRLM were categorized as synchronous (at or up to 30 days after CRC diagnosis to take into account delayed registration), early metachronous (from 1 to 12 months after CRC diagnosis), or late metachronous (more than 12 months after CRC diagnosis), as agreed on in the multisocietal European consensus from 2023.^12^ Other metastases (lung, peritoneal, bone, central nervous system, other [including, but not limited to, distant metastases to the kidney, bladder, bowel, skin, and ovaries], and unspecified) were categorized as synchronous (diagnosed at the time of CRC diagnosis or up to 30 days after) or metachronous (diagnosed more than 30 days after CRC diagnosis).

### Comorbidities, Cancer-Directed Treatment, and Follow-Up

A full list of all registered comorbidities was obtained from the DNPR. This list was used to calculate the Nordic Multimorbidity Index as a composite measure of the overall comorbidity burden for each patient. The Nordic Multimorbidity Index was designed and validated to predict 5-year mortality in a Danish population.^21^ Information on risk factors, such as smoking, alcohol consumption, and body mass index, was obtained from the DCCD.

Cancer-directed treatment for CRLM was ascertained from the DNPR and DPR. We analyzed all treatments recorded up to 30 days before and 180 days after CRLM diagnosis to capture treatment related to the initial management. Treatment was classified as resection, ablation (radiofrequency or microwave), stereotactic body radiation, chemotherapy, targeted therapies, or best supportive care (defined as no record of cancer-directed treatment within the capture period).

For calculation of CRLM incidence, patients were followed up from the date of CRC diagnosis. For calculating survival, patients were followed up from the date of CRLM diagnosis (diagnosed no later than March 8, 2024) until death, emigration, or June 10, 2024, whichever occurred first.

### Statistical Analysis

Descriptive characteristics are presented as means with SDs or medians with IQRs and counts with percentages. We calculated the cumulative incidence of CRLM from date of CRC diagnosis using the Aalen-Johansen estimator to account for death as a competing risk. The association between factors and cumulative incidence was quantified using a multivariable Fine and Gray regression with relative estimates presented as hazard ratios (HRs).^22^ Sensitivity, specificity, positive predictive value, and negative predictive value for CRLM diagnosis in the registries were calculated using a contingency table. Survival analyses were performed using the Kaplan-Meier estimator. For survival analyses, patients were grouped based on year of CRLM diagnosis (2007-2010, 2011-2014, 2015-2019, or 2020-2024). To evaluate variables in overall survival and to examine survival between groups, we performed a multivariable analysis using Cox proportional hazards regression model. The proportional hazards assumption was evaluated by inspection of log-minus-log survival plots and was not violated in any of the analyses. All estimates are presented with associated 95% CIs. Statistical analyses were performed using Stata 18 (StataCorp).

## Results

### Descriptive Characteristics

From the source population of 72 722 patients diagnosed with CRC in Denmark from 2007 to 2021, we identified 14 785 patients with CRLM (median [IQR] age, 68.9 [62.2-77.0] years; 6169 females [41.7%] and 8616 males [58.3%]). Of these patients, 10 188 (68.9%) had synchronous CRLM, 1581 (10.7%) had early metachronous CRLM, and 3016 (20.4%) had late metachronous CRLM. Median (IQR) age at CRLM diagnosis was 70 (62-77) years for synchronous CRLM, 69 (61-76) years for early metachronous CRLM, and 68 (61-75) years for late metachronous CRLM. Most patients were male, and comorbidity levels were similar across groups (Table 1).

**Table 1.** Characteristics of Patients With Colorectal Liver Metastases (N = 14 785)

Characteristic	Patients, No. (%)		
	Synchronous CRLM	Metachronous CRLM	
		Early	Late
All	10 188 (68.9)	1581 (10.7)	3016 (20.4)
Age, median (IQR), y	70 (62-77)	69 (61-76)	68 (61-75)
Age group, y			
<50	572 (5.6)	102 (6.5)	170 (5.6)
50-59	1383 (13.6)	211 (13.3)	456 (15.1)
60-69	3066 (30.1)	497 (31.4)	1026 (34.0)
70-79	3379 (33.2)	536 (33.9)	995 (33.0)
≥80	1788 (17.6)	235 (14.9)	369 (12.2)
Sex			
Female	4366 (42.9)	682 (43.1)	1124 (37.3)
Male	5822 (57.1)	899 (56.9)	1892 (62.7)
Nordic Multimorbidity Index, mean (SD)	7.5 (11.0)	6.8 (10.3)	5.5 (8.7)
Tobacco smoking status			
Nonsmoker	2454 (24.1)	423 (26.8)	895 (29.7)
Current smoker	1387 (13.6)	236 (14.9)	494 (16.4)
Former smoker	2366 (23.2)	440 (27.8)	932 (30.9)
Unknown	3981 (39.1)	482 (30.5)	695 (23.0)
Alcohol consumption, drinks/wk			
0	1793 (17.6)	255 (16.1)	565 (18.7)
1-14	3662 (35.9)	703 (44.5)	1391 (46.1)
>14	825 (8.1)	146 (9.2)	397 (13.2)
Unknown	3908 (38.4)	477 (30.2)	663 (22.0)
T stage			
T1	259 (2.5)	67 (4.2)	174 (5.8)
T2	414 (4.1)	117 (7.4)	319 (10.6)
T3	3512 (34.5)	723 (45.7)	1608 (53.3)
T4	2765 (27.1)	438 (27.7)	630 (20.9)
TX	3238 (31.8)	236 (14.9)	285 (9.4)
N stage			
N0	1406 (13.8)	436 (27.6)	1129 (37.4)
N1	2014 (19.8)	411 (26.0)	842 (27.9)
N2	2750 (27.0)	434 (27.5)	658 (21.8)
N3	156 (1.5)	11 (0.7)	10 (0.3)
NX	3862 (37.9)	289 (18.3)	377 (12.5)
UICC stage			
Stage I	155 (1.5)	133 (8.4)	401 (13.3)
Stage II	306 (3.0)	348 (22.0)	869 (28.8)
Stage III	416 (4.1)	748 (47.3)	1473 (48.8)
Stage IV	8972 (88.1)	234 (14.8)	163 (5.4)
Unknown	339 (3.3)	118 (7.5)	110 (3.6)
BMI mean (SD)	25.2 (4.4)	25.6 (4.6)	26.3 (4.4)
Missing data	3603 (35.4)	469 (29.7)	654 (21.7)
Histologic type			
Adenocarcinoma	8942 (87.8)	1349 (85.3)	2690 (89.2)
Mucinous adenocarcinoma	467 (4.6)	119 (7.5)	230 (7.6)
Signet ring cell carcinoma	44 (0.4)	19 (1.2)	19 (0.6)
Other^a^	154 (1.5)	42 (2.7)	36 (1.2)
Unknown	581 (5.7)	52 (3.3)	41 (1.4)
Tumor location			
Right-sided colon	2574 (25.3)	402 (25.4)	681 (22.6)
Transverse colon	564 (5.5)	82 (5.2)	146 (4.8)
Left-sided colon	3219 (31.6)	453 (28.7)	1019 (33.8)
Rectum	2935 (28.8)	576 (36.4)	1091 (36.2)
Other or unknown^b^	896 (8.8)	68 (4.3)	79 (2.6)
Other synchronous metastases	4471 (43.9)	305 (19.3)	269 (8.9)
Lung	1726 (16.9)	127 (8.0)	143 (4.7)
Peritoneal	946 (9.3)	126 (8.0)	133 (4.4)
Bone	257 (2.5)	13 (0.8)	33 (1.1)
CNS	132 (1.3)	11 (0.7)	25 (0.8)
Unspecified	1071 (10.5)	44 (2.8)	51 (1.7)
Other^c^	372 (3.7)	40 (2.5)	33 (1.1)
Unknown	64 (0.6)	11 (0.7)	≤5^d^
Other metachronous metastases	2459 (24.1)	639 (40.4)	1454 (48.2)
Lung	1267 (12.4)	329 (20.8)	853 (28.3)
Peritoneal	769 (7.5)	180 (11.4)	408 (13.5)
Bone	197 (1.9)	60 (3.8)	148 (4.9)
CNS	132 (1.3)	39 (2.5)	78 (2.6)
Unspecified	563 (5.5)	164 (10.4)	397 (13.2)
Other^c^	335 (3.3)	93 (5.9)	179 (5.9)
Unknown	59 (0.6)	16 (1.0)	52 (1.7)

Abbreviations: BMI, body mass index (calculated as weight in kilograms divided by height in meters squared); CNS, central nervous system; CRLM, colorectal liver metastases; UICC, Union for International Cancer Control.

^a^ Other histologic types include, but not limited to, pleomorphic carcinoma, sarcomatoid carcinoma, and stromal tumor.

^b^ Other tumor site includes diagnosis code DC189: cancer in the colon, not otherwise specified.

^c^ Other synchronous and metachronous metastases include, but not limited to, distant metastases to the kidney, bladder, bowel, skin, and ovaries.

^d^ Exact number not specified to protect the anonymity of patients.

We reviewed 954 medical records of patients with CRC. Of these patients, 250 (26.2%) were diagnosed with CRLM during the follow-up period. Additionally, 175 (70.0%) had synchronous CRLM, 25 (10.0%) had early metachronous CRLM, and 50 (20.0%) had late metachronous CRLM.

### Tumor Characteristics

Among patients with CRLM with a reported location of the primary tumor, left-sided (4691 [34.1%]) and rectal (4602 [33.5%]) tumors were the most common. For patients developing late metachronous CRLM, 1473 (48.8%) had UICC stage III primary CRC. Of patients with synchronous CRLM, 4471 (43.9%) had other synchronous metastases, most commonly in the lungs (1726 [16.9%]) (Table 1).

### Incidence

#### Data From Registries

Based on registry data, the cumulative incidence of CRLM in all patients with CRC was 15.7% (95% CI, 15.4%-15.9%) at 1 year, 18.7% (95% CI, 18.4%-19.0%) at 3 years, and 19.8% (95% CI, 19.5%-20.1%) at 5 years of follow-up. The incidence was highest in male patients (5-year follow-up: 22.2%; 95% CI, 18.6%-26.0%). The median time to metachronous CRLM diagnosis from the date of CRC diagnosis was 482 (95% CI, 390-667) days. The cumulative incidence of CRLM decreased with age but was highest in patients younger than 50 years (5-year follow-up: 27.2%; 95% CI, 25.6%-28.8%). Incidence was also highest in male patients (5-year follow-up: 21.5%; 95% CI, 21.1%-22.0%) (Table 2). The incidence of CRLM was higher in patients with left-sided colon cancer (HR, 1.17; 95% CI, 1.12-1.22) and rectal cancer (HR, 1.05; 95% CI, 1.00-1.09), compared with right-sided colon cancer. The cumulative incidences of CRLM were lower in patients diagnosed with CRC in more recent years (23.1% [95% CI, 22.5%-23.7%] from 2007 to 2010 vs 15.3% [95% CI, 14.7%-15.9%] from 2019 to 2021) (Table 2).

**Table 2.** Cumulative Incidence of Colorectal Liver Metastases at 1, 3, and 5 Years With Associated Hazard Ratios

Variable	Patients, No.	Cumulative incidence of CRLM (95% CI), %			HR (95% CI)
		1 y	3 y	5 y	
All	14 785	15.7 (15.4-15.9)	18.7 (18.4-19.0)	19.8 (19.5-20.1)	NA
Sex					
Female	6166	14.5 (14.1-14.8)	16.2 (15.8-16.6)	17.7 (17.3-18.2)	0.81 (0.79-0.83)
Male	8602	16.7 (16.3-17.1)	19.2 (18.8-19.6)	21.5 (21.1-22.0)	1 [Reference]
Age, y					
<50	840	21.5 (20.0-23.0)	24.4 (22.9-25.9)	27.2 (25.6-28.8)	1.21 (1.12-1.30)
50-59	2048	18.4 (17.6-19.3)	21.5 (20.6-22.4)	24.0 (23.1-24.9)	1.05 (0.99-1.11)
60-69	4584	17.7 (17.2-18.2)	20.2 (19.6-20.8)	22.7 (22.1-23.3)	1 [Reference]
70-79	4904	15.4 (14.9-15.8)	17.5 (17.0-18.0)	19.3 (18.9-19.8)	0.83 (0.79-0.86)
≥80	2392	11.5 (11.0-12.0)	12.7 (12.2-13.2)	13.7 (13.2-14.2)	0.56 (0.53-0.59)
Tumor location					
Right-sided colon	3655	14.9 (14.4-15.4)	16.8 (16.3-17.3)	18.4 (17.9-19.0)	1 [Reference]
Transverse colon	790	14.7 (13.7-15.8)	16.5 (15.4-17.7)	18.2 (17.0-19.4)	0.98 (0.91-1.06)
Left-sided colon	4689	16.5 (16.0-17.0)	18.9 (18.4-19.5)	21.1 (20.6-21.7)	1.17 (1.12-1.22)
Rectum	4594	14.6 (14.1-15.2)	17.0 (16.6-17.5)	19.2 (18.7-19.7)	1.05 (1.00-1.09)
Unknown	1040	21.1 (19.9-22.3)	22.1 (20.9-23.3)	23.0 (21.8-24.2)	1.30 (1.21-1.39)
Year of CRC diagnosis					
2007-2010	4265	18.1 (17.5-18.7)	20.5 (19.9-21.1)	23.1 (22.5-23.7)	1 [Reference]
2011-2014	4381	17.0 (16.5-17.6)	19.4 (18.9-20.0)	21.6 (20.9-22.1)	0.91 (0.88-0.95)
2015-2018	4002	14.4 (13.9-14.9)	16.4 (15.9-16.8)	18.2 (17.7-18.7)	0.76 (0.72-0.79)
2019-2021	2120	12.6 (12.1-13.2)	14.3 (13.7-14.9)	15.3 (14.7-15.9)	0.63 (0.60-0.66)
UICC stage at CRC diagnosis					
Stage I	688	2.3 (2.0-2.5)	3.3 (3.0-3.6)	4.8 (4.4-5.2)	0.38 (0.35-0.42)
Stage II	1523	3.7 (3.4-4.0)	5.8 (5.4-6.1)	7.8 (7.4-8.2)	0.62 (0.58-0.66)
Stage III	2633	6.0 (5.7-6.3)	9.7 (9.3-10.1)	13.0 (12.5-13.5)	1 [Reference]
Unknown	563	7.3 (6.6-7.9)	7.9 (7.3-8.6)	8.7 (8.0-9.4)	0.65 (0.60-0.72)

Abbreviations: CRC, colorectal cancer; CRLM, colorectal liver metastases; HR, hazard ratio; NA, not applicable; UICC, Union for International Cancer Control.

#### Data From Medical Records

**Figure 1.**
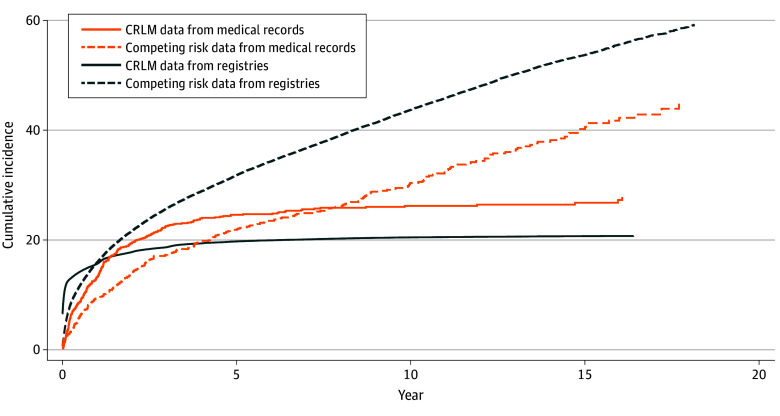
Cumulative Incidence of Colorectal Liver Metastases (CRLM) and Competing Risk of Death Data From Medical Records and Danish National Registries

The cumulative incidence of CRLM was 13.3% (95% CI, 11.2%-15.5%) at 1 year, 22.6% (95% CI, 20.4%-25.3%) at 3 years, and 24.7% (95% CI, 22.0%-27.5%) at 5 years of follow-up (Figure 1). The median time to a diagnosis of metachronous CRLM from the date of CRC diagnosis was 455 (95% CI, 460-503) days.

Among the 954 patients in the medical records review sample, 294 (30.8%) had a diagnosis of CRLM in the registries. Of these patients, 250 were true positives (defined as patients with CRLM in both registries and medical records). In addition, 778 patients had no record of CRLM in the registries, of whom 704 (90.5%) were true negatives (defined as patients without CRLM in both registries and medical records). The sensitivity was 77.2% (95% CI, 72.6%-81.8%), and the specificity was 94.4% (95% CI, 92.7%-96.0%). Positive and negative predictive values were 85.6% (95% CI, 81.6%-89.6%) and 90.5% (95% CI, 88.4%-92.5%), respectively (eTable in Supplement 1).

### Treatment

**Figure 2.**
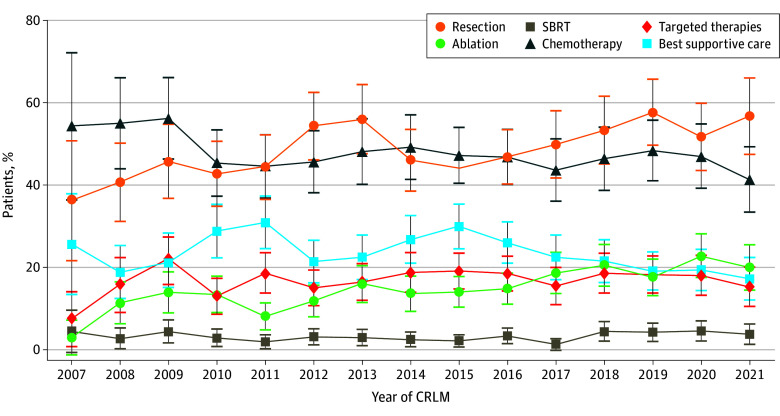
Percentage of Patients With Colorectal Liver Metastases (CRLM) Receiving Different Treatments, From 2007 to 2022 SBRT indicates stereotactic body radiation. Error bars represent 95% CIs.

Throughout the study, patients underwent surgery (5798 [39.2%]), ablation (2062 [13.9%]), stereotactic body radiation (340 [2.3%]), chemotherapy (8699 [58.4%]), and targeted therapies with antibodies against the epidermal growth factor receptor or vascular endothelial growth factor receptor (3694 [25.0%]). Furthermore, 3424 patients (23.2%) were considered unfit for active treatment and received best supportive care. Except for an increased use of ablation (from 3.8% in 2007 to 21.2% in 2021), the choice of treatment modality was relatively stable throughout the study (Figure 2). For patients diagnosed with synchronous CRLM, between 58% and 67% were treated with chemotherapy, and less than 40% underwent surgical resection. This finding was in contrast to that in patients with early and late CRLM, more than 40% of whom underwent surgery and less than 60% received chemotherapy (eFigures 2-4 in Supplement 1).

### Survival

Throughout the study period, 1-year survival increased for patients with synchronous CRLM from 47.2% (95% CI, 45.3%-49.1%) in 2007 to 2010 to 58.3% (95% CI, 54.1%-62.3%) in 2020 to 2024. For early and late metachronous CRLM, no improvement in 1-year survival was observed (Table 3). Survival was highest for early synchronous CRLM throughout the study (eFigure 5 in Supplement 1). Similarly, 5-year survival increased for patients with synchronous CRLM from 13.0% (95% CI, 11.7%-14.3%) in 2007 to 2010 to 20.7% (95% CI, 18.1%-23.5%) in 2020 to 2024 (Table 3).

**Table 3.** Survival in Colorectal Liver Metastases by Diagnosis Period

	CRLM diagnosis period			
	2007-2010	2011-2014	2015-2019	2020-2024
**CRLM survival (95% CI), %**				
1 y	50.4 (48.7-52.1)	55.9 (54.4-57.4)	58.3 (56.9-59.7)	60.5 (58.5-62.6)
3 y	24.5 (23.1-26.0)	30.8 (29.5-32.2)	31.0 (29.7-32.3)	33.6 (31.5-35.7)
5 y	16.2 (15.0-17.5)	20.1 (19.4-21.8)	20.9 (19.7-22.0)	24.1 (21.8-26.3)
Median (95% CI), y	1.0 (0.9-1.1)	1.3 (1.2-1.4)	1.4 (1.3-1.5)	1.5 (1.4-1.7)
**Synchronous CRLM survival (95% CI), %**				
1 y	47.1 (45.2-49.0)	53.3 (51.5-55.0)	56.5 (54.8-58.2)	57.4 (54.4-60.3)
3 y	20.8 (19.3-22.3)	27.3 (25.8-29.0)	27.5 (26.0-29.0)	29.3 (26.6-32.0)
5 y	13.0 (11.7-14.3)	17.5 (16.2-19.0)	18.4 (17.1-19.8)	20.7 (18.1-23.5)
Median (95% CI), y	0.9 (0.8-1.0)	1.2 (1.0-1.3)	1.3 (1.2-1.5)	1.3 (1.2-1.5)
**Early metachronous CRLM survival (95% CI), %**				
1 y	64.6 (59.9-68.9)	69.2 (64.5-73.4)	65.0 (60.8-68.9)	70.7 (63.1-77.1)
3 y	39.3 (34.7-43.9)	45.1 (40.2-49.8)	40.0 (35.9-44.1)	45.7 (37.9-53.1)
5 y	28.7 (24.6-33.0)	33.7 (29.2-38.3)	26.5 (22.8-30.3)	28.4 (20.6-36.7)
Median (95% CI), y	2.0 (1.7-2.4)	2.4 (2.0-2.9)	1.9 (1.6-2.4)	2.4 (1.6-3.3)
**Late metachronous CRLM survival (95% CI), %**				
1 y	59.1 (53.0-64.6)	59.3 (55.7-62.6)	60.5 (57.6-63.2)	62.7 (59.1-66.0)
3 y	36.6 (30.1-42.3)	37.1 (33.7-40.4)	36.8 (34.1-39.6)	37.6 (34.0-41.2)
5 y	27.2 (22.1-32.5)	25.6 (22.6-28.7)	25.1 (22.6-27.6)	27.8 (24.1-31.6)
Median (95% CI), y	1.6 (1.2-2.0)	1.7 (1.5-1.9)	1.7 (1.5-1.9)	1.8 (1.5-2.0)

Abbreviation: CRLM, colorectal liver metastases.

Across all liver metastases, the outcome for patients with right-sided colon cancer was worse than for patients with left-sided (HR, 0.83; 95% CI, 0.79-0.87) and rectal (HR, 0.81; 95% CI, 0.77-0.85) primary tumors. After adjustment for sex, age, tumor location of primary, and year of CRC diagnosis, both synchronous CRLM (HR, 1.46; 95% CI, 1.38-1.55) and late metachronous CRLM (HR, 1.17; 95% CI, 1.09-1.25) were associated with worse outcomes compared with early metachronous CRLM.

## Discussion

In this nationwide study, we observed increased survival among patients with CRLM diagnosed within the first year of CRC diagnosis (ie, synchronous and early metachronous) and a decreasing cumulative incidence over time for all CRLM types. The declining cumulative incidence of CRLM may partly be attributed to a more structured and expedited approach to cancer treatment initiation in Denmark, better organization of multidisciplinary team conferences, and the introduction of a national CRC screening program in 2014. CRC screening programs have been shown to lead to diagnosis at earlier tumor stages.^23,24^

The cumulative incidence of CRLM was highest in patients younger than 50 years (27.2% at 5 years). This finding aligns with previous reports of an increasing incidence of CRC among younger adults.^25,26,27^ The increased risk of CRLM in younger patients may be associated with diet and lifestyle factors as well as a potentially more aggressive tumor biology and thus a greater propensity for early tumor dissemination.^28^ Higher risk may also in part be attributable to insufficient attention to the cancer risk in younger adults, which may lead to later diagnosis at more advanced stages, as younger adults are generally not regarded as a high-risk group for malignant neoplasms.

In the Danish national registries, we observed a 5-year cumulative incidence of approximately 20%, which is lower than the estimates reported in previous international studies.^3,4^ It should be acknowledged that patients diagnosed in 2021 had not yet accumulated 5 years of follow-up at the time of analysis, which may have contributed to a reduction in cumulative incidence. In contrast, the incidence we identified through manual medical record review was 26.2%, which aligns more closely with existing literature.^29^ The discrepancy between registry-based and clinically verified data may reflect the incomplete registration of CRLM in subgroups such as older (≥65 years), frail patients and those with rapidly progressive disease, for whom metastatic progression may not be systematically documented. Nevertheless, registry capture of patients receiving active treatment for CRLM is assumed to be high due to the association between treatment registration and hospital reimbursement within the Danish health care system.

The 1-year survival for patients with synchronous CRLM increased approximately 10 percentage points but was consistent for patients with early and late metachronous CRLM throughout the study. Furthermore, no improvement in 5-year survival was observed for patients with late metachronous CRLM. The general increase in survival may be attributed to a more aggressive treatment approach, particularly surgical management of liver metastases. Over time, surgical strategies have evolved to include larger resections, 2-stage procedures, and a more accurate diagnosis of all metastases in the liver.^30^ In addition, the use of radiofrequency ablation has increased, and the precision of this technique has improved drastically.^31,32^ For patients with CRC, assessment of mutational status has been standard in Denmark since 2013, leading to a more precise and more personalized oncological treatment with targeted therapies.

No improvement in 5-year survival was observed in patients with metachronous CRLM, which is consistent with findings from other studies.^11^ This finding may reflect underlying biological differences between metachronous and synchronous disease, with some studies suggesting a more indolent but also more treatment-resistant progression.^33^ We observed higher survival in patients with early metachronous CRLM compared with late metachronous CRLM, which may be attributable to a higher burden of comorbidity and older age in the latter group, possibly leading to less-intensive treatment strategies. Additionally, the prevalence of other distant metastases was more common in late metachronous CRLM compared with early metachronous CRLM.

### Strengths and Limitations

This study had strengths. The collection of data in a uniform, tax-financed health care system in combination with mandatory reporting of all cancers and a virtually complete follow-up contributed to the high validity of our findings. The nationwide population-based study design ensured a high degree of generalizability to the Danish population with CRC. However, differences in treatment options and health care organizations may limit the transferability of these results.

The findings provide important insights into temporal patterns of incidence and survival among patients with CRLM in Denmark. However, these results should be interpreted with consideration of several limitations. First, incomplete registration of metastatic disease likely led to an underestimation of the cumulative incidence of CRLM, as suggested by the higher incidence observed in the medical record review.^34,35,36^ Second, limited follow-up for patients diagnosed in recent years may have reduced cumulative incidence and survival estimates. Third, residual confounding by comorbidity, frailty, and treatment intent cannot be excluded. Finally, given that it is an observational study, causal inferences cannot be drawn.

## Conclusions

The cumulative incidence of CRLM decreased during the study across all age groups, with the highest cumulative incidence among patients younger than 50 years. Survival increased over time for patients with synchronous CRLM and early metachronous CRLM, but no improvement in survival was observed for patients with late metachronous CRLM. A manual medical record review suggested an underreporting of CRLM in the Danish national registries. These findings may reflect differences in tumor biology, comorbidity burden, and treatment eligibility between metachronous and synchronous CRLM and may support targeted follow-up and treatment strategies based on the timing of metastasis.

## Notes

Supplement 1. **eTable.** Contingency Table Presenting the Validation of a CRLM Diagnosis in the Danish National Registries **eFigure 1.** Overview of Registries Used, and Selection Criteria Applied to Create the Population **eFigure 2.** Percentage With 95% CI of Patients With sCRLM Treated With: Surgery, Ablation, Chemotherapy, Radiation Therapy, Targeted Treatment, and Best Supportive Care in Denmark From 2007 to 2022 **eFigure 3.** Percentage With 95% CI of Patients With Early mCRLM Treated With: Surgery, Ablation, Chemotherapy, Radiation Therapy, Targeted Treatment, and Best Supportive Care in Denmark From 2007 to 2022 **eFigure 4.** Percentage With 95% CI of Patients With Late mCRLM Treated With: Surgery, Ablation, Chemotherapy, Radiation Therapy, Targeted Treatment, and Best Supportive Care in Denmark From 2007 to 2022 **eFigure 5.** Kaplan-Meier Survival Estimates of Patients With sCRLM, Early mCRLM, and Late mCRLM in Denmark Diagnosed From 2007 to 2024 With 95% CI

Supplement 2.
Data Sharing Statement
