# Supplementary material for: Liver Metastases and Survival Among Patients With Colorectal Cancer
Source: JAMA Netw Open. 2025 Dec 22;8(12):e2550467. doi: 10.1001/jamanetworkopen.2025.50467 (PMC12723549; doi:10.1001/jamanetworkopen.2025.50467)
Supplement: Supplement 1. — eTable. Contingency Table Presenting the Validation of a CRLM Diagnosis in the Danish National Registries eFigure 1. Overview of Registries Used, and Selection Criteria Applied to Create the Population eFigure 2. Percentage With 95% CI of Patients With sCRLM Treated With: Surgery, Ablation, Chemotherapy, Radiation Therapy, Targeted Treatment, and Best Supportive Care in Denmark From 2007 to 2022 eFigure 3. Percentage With 95% CI of Patients With Early mCRLM Treated With: Surgery, Ablation, Chemotherapy, Radiation Therapy, Targeted Treatment, and Best Supportive Care in Denmark From 2007 to 2022 eFigure 4. Percentage With 95% CI of Patients With Late mCRLM Treated With: Surgery, Ablation, Chemotherapy, Radiation Therapy, Targeted Treatment, and Best Supportive Care in Denmark From 2007 to 2022 eFigure 5. Kaplan-Meier Survival Estimates of Patients With sCRLM, Early mCRLM, and Late mCRLM in Denmark Diagnosed From 2007 to 2024 With 95% CI [file jamanetwopen-e2550467-s001.pdf]

Supplemental Online Content

Johannsen IR, Boysen AK, Nielsen MF, Mortensen FV, Kirkegård J. Liver metastases and survival among patients with colorectal cancer. *JAMA Netw Open*. 2025;8(12):e2550467. doi:10.1001/jamanetworkopen.2025.50467

eTable. Contingency Table Presenting the Validation of a CRLM Diagnosis in the Danish National Registries

eFigure 1. Overview of Registries Used, and Selection Criteria Applied to Create the Population

eFigure 2. Percentage With 95% CI of Patients With sCRLM Treated With: Surgery, Ablation, Chemotherapy, Radiation Therapy, Targeted Treatment, and Best Supportive Care in Denmark From 2007 to 2022

eFigure 3. Percentage With 95% CI of Patients With Early mCRLM Treated With: Surgery, Ablation, Chemotherapy, Radiation Therapy, Targeted Treatment, and Best Supportive Care in Denmark From 2007 to 2022

eFigure 4. Percentage With 95% CI of Patients With Late mCRLM Treated With: Surgery, Ablation, Chemotherapy, Radiation Therapy, Targeted Treatment, and Best Supportive Care in Denmark From 2007 to 2022

eFigure 5. Kaplan-Meier Survival Estimates of Patients With sCRLM, Early mCRLM, and Late mCRLM in Denmark Diagnosed From 2007 to 2024 With 95% CI

This supplemental material has been provided by the authors to give readers additional information about their work.

© 2025 Johannsen IR et al. *JAMA Network Open*

eTable 1. Contingency table presenting the validation of a CRLM diagnosis in the Danish national registries.

		Medical record review	
		**CRLM**	**No CRLM**
**Registries**	**CRLM**	250	42
	**No CRLM**	74	704
Sensitivity = 250/324 = 77.2 % (95% CI: 72.6% - 81.8%)<br>Specificity = 704/746 = 94.4 % (95% CI: 92.7% - 96.0%)<br>PPV = 250/292 = 85.6 % (95% CI: 81.6% - 89.6%)<br>NPV = 704/778 = 90.5 % (95% CI: 88.4% - 92.5%)			

Contingency table presenting the validation of a CRLM diagnosis in the Danish national registries.

Abbreviations: PPV, Positive predictive value; NPV, negative predictive value.

© 2025 Johannsen IR et al. *JAMA Network Open*

eFigure 1. Overview of registries used, and selection criteria applied to create the population

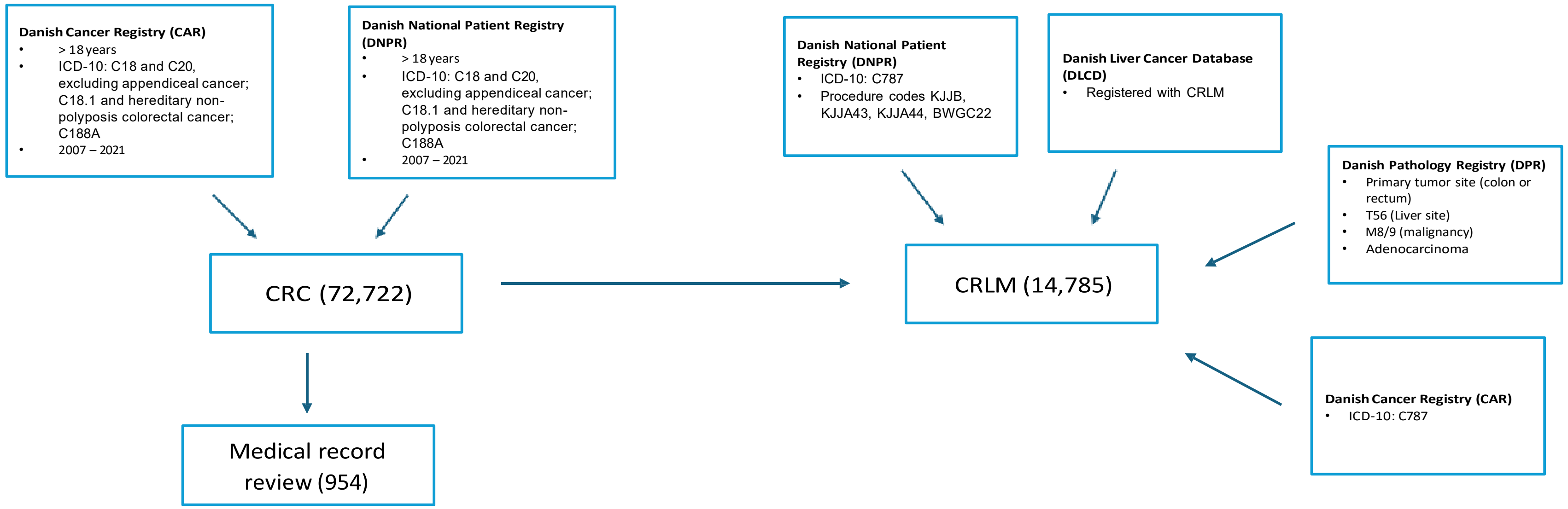

© 2025 Johannsen IR et al. *JAMA Network Open*

eFigure 2. Percentage with 95% CI of patients with sCRLM treated with: surgery, ablation, chemotherapy, radiation therapy, targeted treatment, and best supportive care in Denmark from 2007 to 2022.

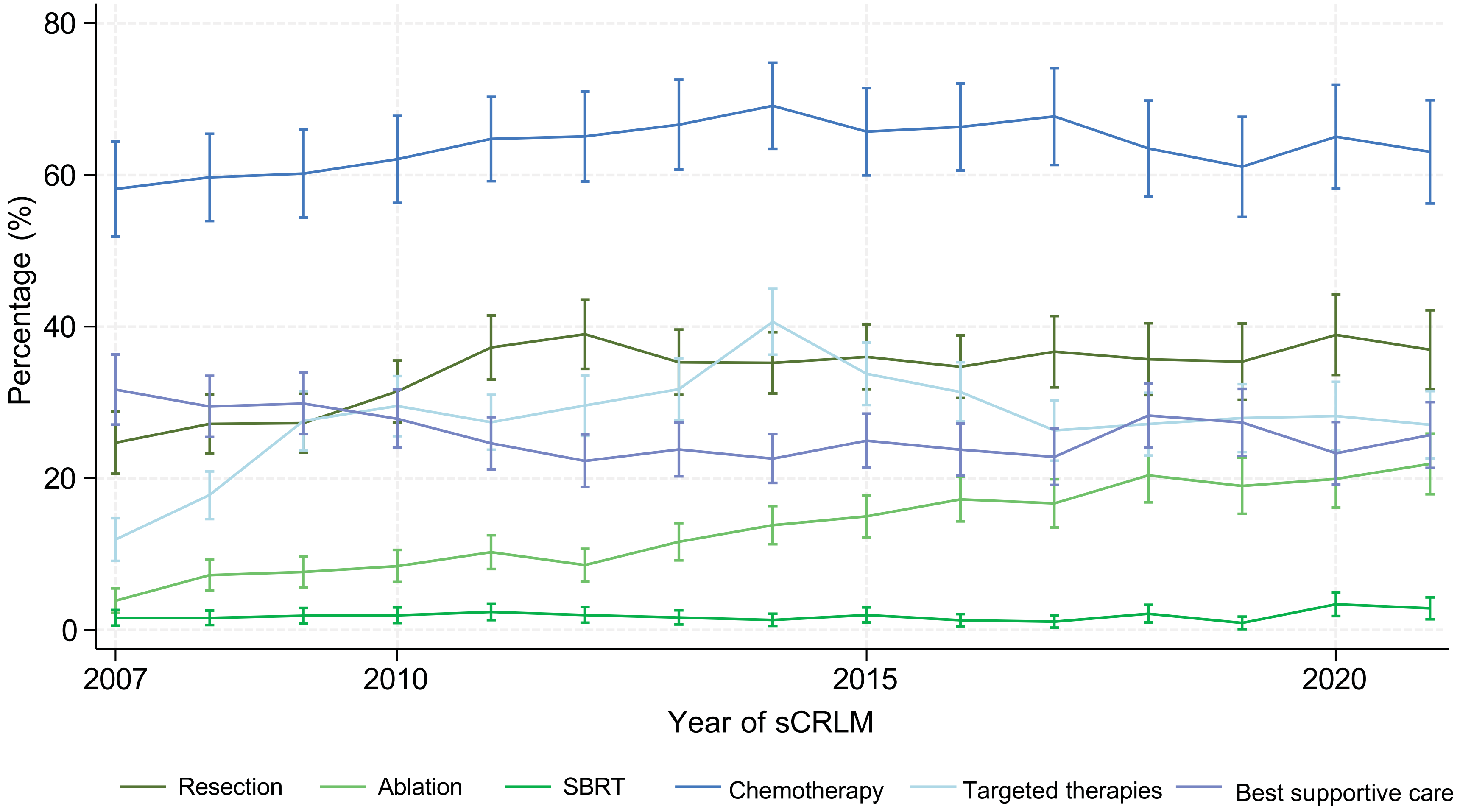

© 2025 Johannsen IR et al. *JAMA Network Open*

eFigure 3. Percentage with 95% CI of patients with early mCRLM treated with: surgery, ablation, chemotherapy, radiation therapy, targeted treatment, and best supportive care in Denmark from 2007 to 2022.

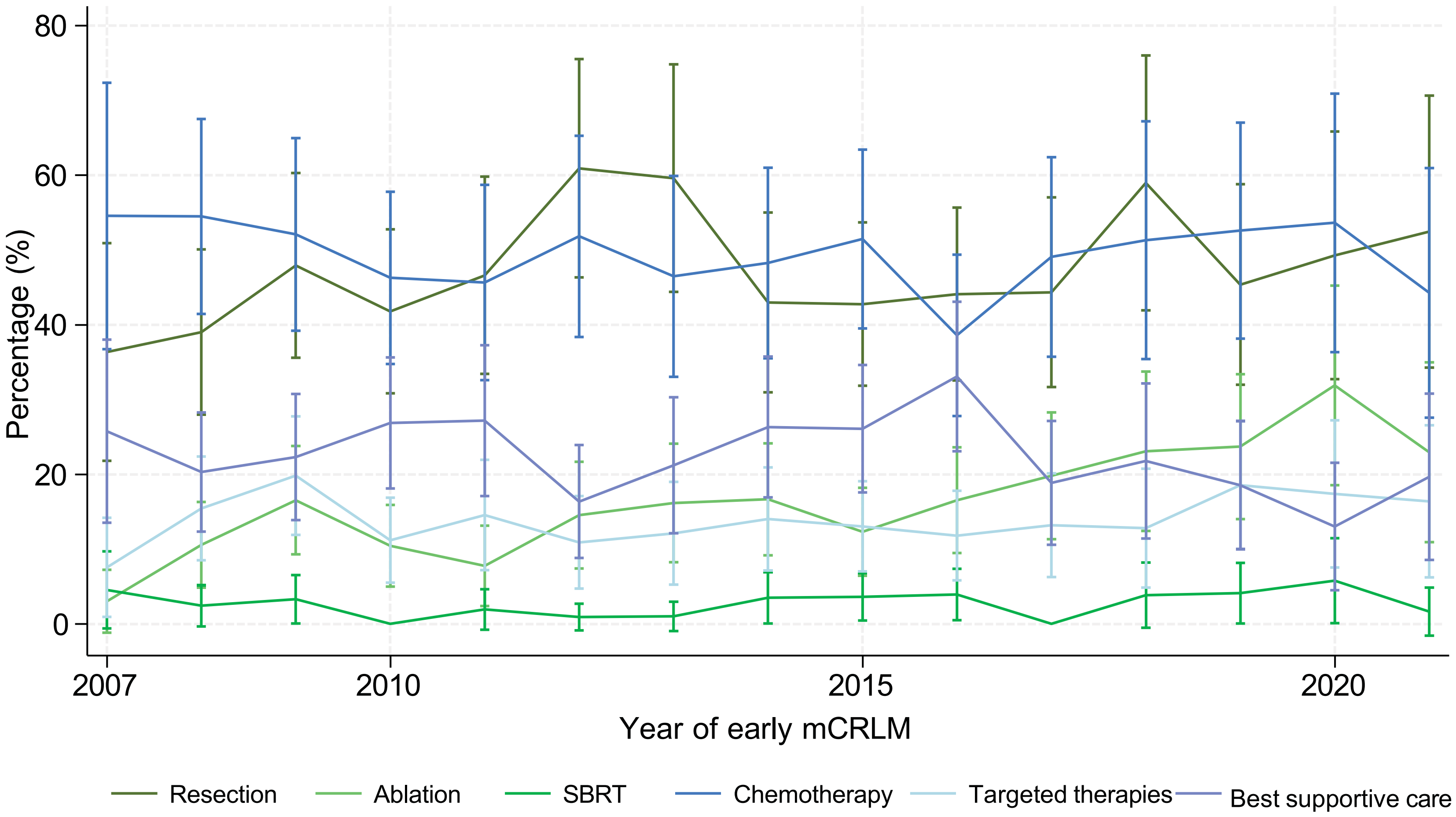

© 2025 Johannsen IR et al. *JAMA Network Open*

eFigure 4. Percentage with 95% CI of patients with late mCRLM treated with: surgery, ablation, chemotherapy, radiation therapy, targeted treatment, and best supportive care in Denmark from 2007 to 2022.

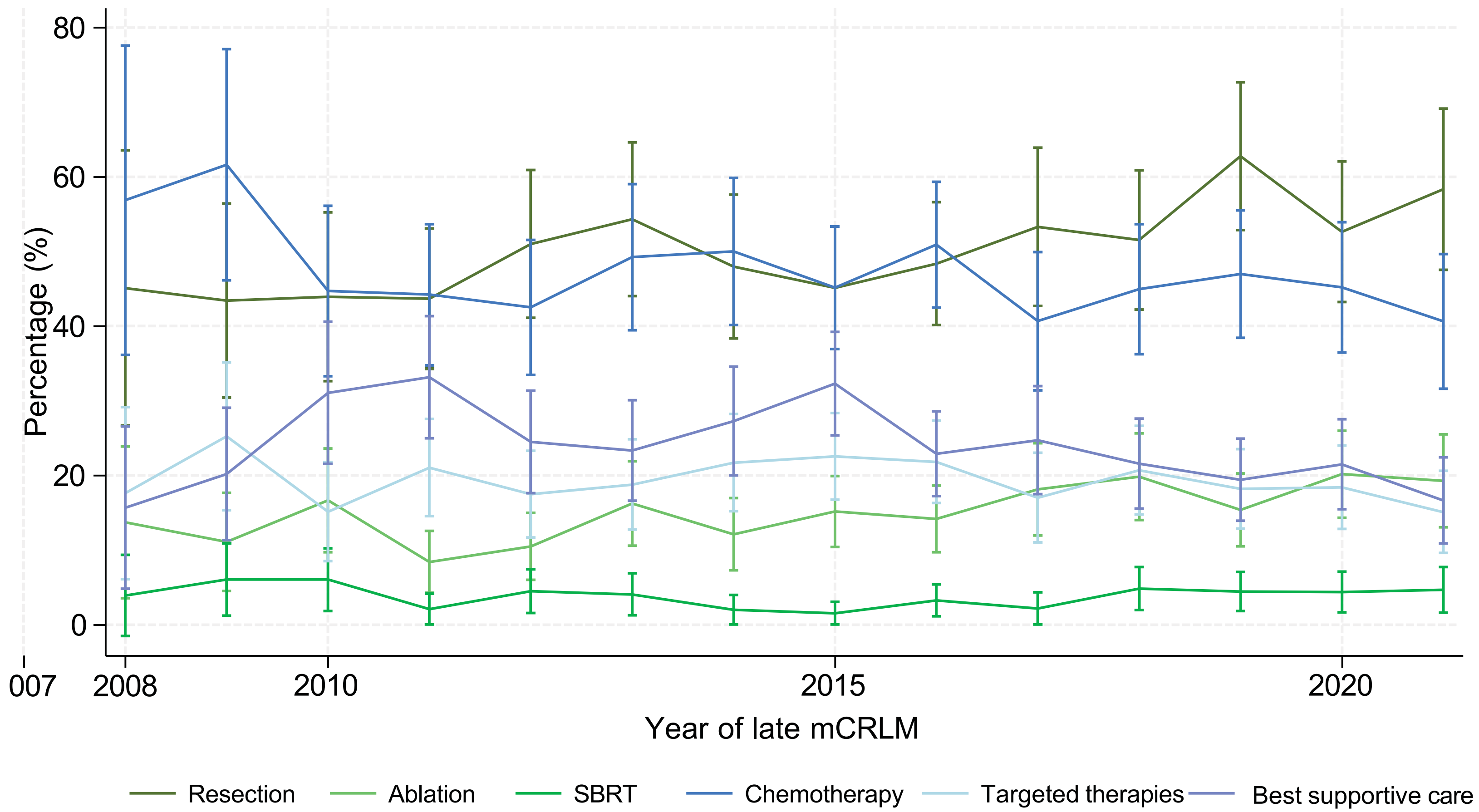

© 2025 Johannsen IR et al. *JAMA Network Open*

eFigure 5. Kaplan-Meier survival estimates of patients with sCRLM, early mCRLM, and late mCRLM in Denmark diagnosed from 2007 to 2024 with 95% CI.

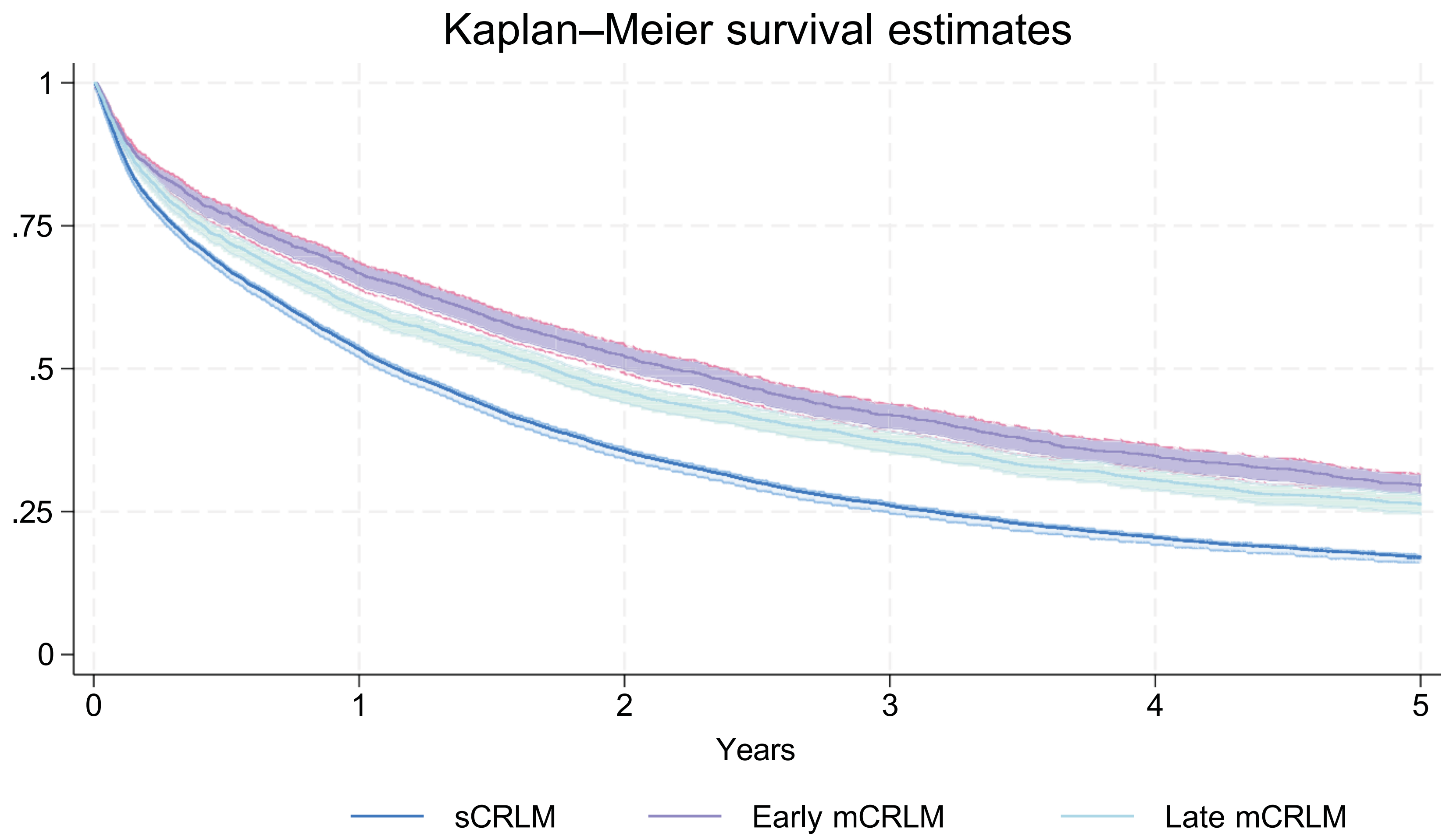

© 2025 Johannsen IR et al. *JAMA Network Open*
