# Supplementary material for: Liver Metastases and Survival Among Patients With Colorectal Cancer
Source: JAMA Netw Open. 2025 Dec 22;8(12):e2550467. doi: 10.1001/jamanetworkopen.2025.50467 (PMC12723549; doi:10.1001/jamanetworkopen.2025.50467)
Supplement: Supplement 2. — Data Sharing Statement [file jamanetwopen-e2550467-s002.pdf]

Data Sharing Statement

Johannsen. Liver Metastases and Survival Among Patients With Colorectal Cancer. *JAMA Netw Open*. Published December 22, 2025. doi:10.1001/jamanetworkopen.2025.50467

Data
Data available: No

Additional Information
Explanation for why data not available: The data underlying this manuscript cannot be shared without violation of Danish law. Research can obtain access to the data through application at the Danish Health Data Authority (www.sundhedsdatastyrelsen.dk).
